# Coliform and Metal Contamination in Lago de Colina, a Recreational Water Body in Chihuahua State, Mexico

**DOI:** 10.3390/ijerph8062386

**Published:** 2011-06-23

**Authors:** Hector Rubio-Arias, Nora I. Rey, Rey M. Quintana, G. Virginia Nevarez, Oskar Palacios

**Affiliations:** 1 Autonomous University of Chihuahua, Periferico Francisco R. Almada, Km. 1 Colonia Zootecnia, Chihuahua, Chih. C.P. 31000, Mexico; E-Mails: rquintan@uach.mx (R.M.Q.); vnevare@uach.mx (G.V.N.); 2 Department of Natural Resources, College of Zoo-technology and Ecology, Autonomous University of Chihuahua, Periferico Francisco R. Almada, Km. 1 Colonia Zootecnia, Chihuahua, Chih. C.P.31453, Mexico; E-Mails: ivet_rey77@hotmail.com (N.I.R.); opalacios@hotmail.com (O.P.)

**Keywords:** heavy metals, pollution, body water, recreational areas, Chihuahua, Mexico

## Abstract

Lago de Colina (Colina Lake) is located about 180 km south of the city of Chihuahua (Mexico), and during the Semana Santa (Holy Week) vacation period its recreational use is high. The objective of this study was to quantify coliform and heavy metal levels in this water body before and after the Holy Week vacation period in 2010. Twenty sampling points were randomly selected and two water samples were collected at each point near the surface (0.30 m) and at 1 m depth. After the Holy Week vacation the same twenty points were sampled at the same depths. Therefore, a total 80 water samples were analyzed for fecal and total coliforms and levels of the following metals: Al, As, B, Ca, Cr, Cu, Fe, K, Mg, Mn, Na, Ni, Pb, Se, Si and Zn. It was hypothesized that domestic tourism contaminated this water body, and as a consequence, could have a negative impact on visitor health. An analysis of variance (ANOVA) study was performed for each element and its interactions considering a factorial design where factor A was sample date and factor B was sample depth. Fecal coliforms were only detected at eight sampling points in the first week, but after Holy Week, both fecal and total coliforms were detected at most sampling points. The concentrations of Al, B, Na, Ni and Se were only statistically different for factor A. The levels of Cr, Cu, K and Mg was different for both date and depth, but the dual factor interaction was not significant. The amount of Ca and Zn was statistically different due to date, depth and their interaction. No significant differences were found for any factor or the interaction for the elements As, Fe and Mn. Because of the consistent results, it is concluded that local tourism is contaminating the recreational area of Colina Lake, Chihuahua, Mexico.

## 1. Introduction

The Mexican government is beginning to promote domestic tourism throughout Mexico as a means to generate new jobs and to improve local economies. One of the main problems in the Mexican beaches or coastal areas used as recreational areas is the level of water contamination [1–3]. Mexico’s economy as a whole depends largely on international tourism, especially in those areas which have recreational beaches. However, in rural areas it is difficult to promote this sort of tourism; therefore, domestic tourism should be incentivized. The problem is aggravated in Mexican states that do not have good aquatic resources or where access to aquatic recreational areas is difficult and expensive.

Chihuahua is the largest state in Mexico and is located in the north central region, just south of the US-Mexico border. The overall environment in Chihuahua is arid or semi-arid and, as a consequence, there are few aquatic resources, making it difficult to promote local tourism because it is generally well known that mountain scenarios rank second after coastal regions as popular tourist destinations [4]. Colina Lake is a man-made water reservoir where local inhabitants depend on domestic tourism that comes mostly from the city of Chihuahua, which has about one million inhabitants. Moreover, Chihuahua’s stakeholders depend on Colina Lake for commercial purposes and fishing activities, as well as for recreational purposes. For example, it is estimated that during the Semana Santa (Holy Week) period the lake receives 25,000 to 30,000 visitors [5]. Holy Week is a religious-based festivity that lasts one week and extends to all Mexico. It is important to point out that Chihuahua’s tourism department and other local agencies have aggressively promoted Colina Lake as a recreational destination.

After the Holy Week vacation period, the lake is notorious for having large amounts of trash in the area, and reception of discharged domestic wastewaters and solid waste dumping and therefore, we hypothesized that the water would be contaminated, creating a dangerous conditions for visitors as well as for the local inhabitants in the Colina Lake area and that consequently the viability of the recreational center might be in jeopardy. Consequently, the objective of this study was to quantify fecal and heavy metal levels in the water of the Colina Lake before and after the Holy Week vacation period. These results will be important for the different levels of Chihuahua’s authorities to promote future conservation actions for this aquatic resource to have a clean environment; otherwise it will be undermined as one of the state’s major economic tourist forces.

## 2. Materials and Methods

**Figure 1.**
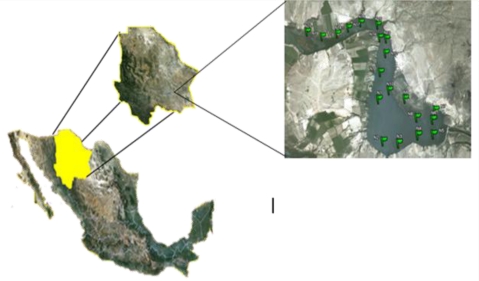
Map showing Mexico, the State of Chihuahua and the sampling points around Colina Lake.

The study was conducted during 2010 and carried out at Lago de Colina (Colina Lake) located in the municipality of San Francisco de Conchos of the State of Chihuahua, Mexico (Figure 1). This location is about 180 km south of the city of Chihuahua (27° 34′ 38″ North latitude; and 105° 23′ 48″ West longitude), and the lake is about 3 km wide and 8 km long. The main water source is the Conchos River and the lake’s depth is 25–35 m. This water reservoir is a man-made lake resulting from the impoundment of the Conchos River by the La Boquilla dam, and is considered the biggest one in Chihuahua, with a capacity of 2,895 Mm^3^ [6]. The maximum ambient temperature is 41.7 °C, and the minimum is 14.1 °C. Average annual precipitation is 363 mm. and the dam is about 1,250 meters above sea level. The lake was divided using satellite imagery and then 20 sampling locations were randomly selected and properly georeferenced. The 20 locations were sampled on March 21 before the Holy Week vacation period of 2010 and then the same locations were sampled again on April 15 after the vacation period. At each point, four water samples were obtained – two in the top 0.30 m and two at 1.0 m depth. Of the two top samples, one was used to quantify coliform presence and the other was used to measure physico-chemical and metal level variables. The same was done with the two samples obtained at 1.0 depth. The water samples were obtained following procedures established in the Mexican norm NMX-AA-014-1980 [7].

Temperature, pH and electrical conductivity (EC) values were determined *in situ*. The temperature was recorded using a mercury thermometer according to the Mexican norm NMX-AA-007-SCF [8]. The pH was measured with a Hanna Waterproof pH/EC/Temp potentiometer instrument according to the Mexican norm NMX-AA-008-SCF [9]. The EC was obtained with the same device used to determine the pH according to the Mexican norm NMX-AA-093-SCF [10]. The water samples were collected in 1 L sterilized containers and properly preserved in a cool place (4 °C). Immediately after the water sample was obtained, a 1 L sample was transported to the laboratory of the College of Chemistry of the Autonomous University of Chihuahua to evaluate total and fecal coliforms. The other 1 L samples were transported to the laboratory of the Faculty of Zootechnology and Ecology of the Autonomous University of Chihuahua for further analysis.

The evaluated metals were: aluminum (Al), arsenic (As), boron (B), calcium (Ca), chromium (Cr), copper (Cu), iron (Fe), potassium (K), magnesium (Mg), manganese (Mn), sodium (Na), nickel (Ni), lead (Pb), selenium (Se), silicon (Si) and zinc (Zn). Metals were quantified using a Perkin Elmer 2100 Inductively Coupled Plasma-Optical Emission Spectrometer (ICP-OES) property of La Campana Experimental Station (which is part of the Mexican National Research Institute for Forestry, Agriculture and Animal Production) after a sample digestion performed according to the Mexican norm NMX-AA-051-SCFI [11] as follows: a portion of each sample (100 mL) was filtered using a Whatman filter paper and digested completely with 5 mL of concentrated HNO_3_. For the quantification of the different elements, standards of known concentrations were prepared for each one, followed by suitable calibration of the wavelength, plasma position and gas flux. The fecal and total coliforms were quantified according to the following procedure: a 100 mL water sample was filtered through a 0.45 μm porosity membrane utilizing a Kitasato flask connected to a vacuum system. The sample was then placed in red-violet agar (RVA) for contact of the membrane with the agar plat and the samples were then incubated for 24 h at 35 ±0.5 °C for total coliforms and 44.5 °C for fecal coliforms. Finally, Colony Forming Units (CFU) per 100 mL of water were counted [12]. All tests were done in duplicate. The sample means of coliforms were then transformed to logarithmic units (log) to compare the results with those established in the Mexican Norm NOM 112-SSA1-1994 [13].

## 3. Results and Discussion

In general, there were no significant differences in water samples concerning pH, temperature and electrical conductivity. Nevertheless, an increase in these parameters was noted after the Holy Week vacation period (Table 1).

Over 22% of the water samples tested positive for total coliforms before the Holy Week vacation sampling period (Table 2 format all tables the same way). It is important to point out that no fecal coliforms were detected in any water sample before the Holy Week sampling. In contrast, most of the water samples after the Holy Week period contained total coliforms (Table 3). In addition, fecal coliforms were detected in about 45% of total water samples after Holy Week (Table 4). Coliform presence is an excellent variable and widely utilized to establish water quality in terms of microbiological contamination [14]. In this particular study, it is apparent that human activity due to domestic tourism is contaminating this recreational aquatic area and is, therefore, a potential public health threat. In fact, according to the Health Department of the Chihuahua’s State Government gastrointestinal illnesses represent the highest reported illness for visitors to Colina Lake during the Holy Week vacation period.

From a total of 18 metals originally considered in the present study, Ag was not detected and the following metals were not statistically analyzed because of inconsistency of results: As, Cd, Fe, Se and Mn. In general, the concentrations of Al, B, Na, Ni and Se were statistically different according to factor A (sampling date-before and after) while the levels of Cr, Cu, K and Mg were statistically different due to factor A as well as factor B (depth) but no interaction was detected. The elements Ca and Zn were statistically different due to factor A, factor B, and their interaction.

**Figure 2.**
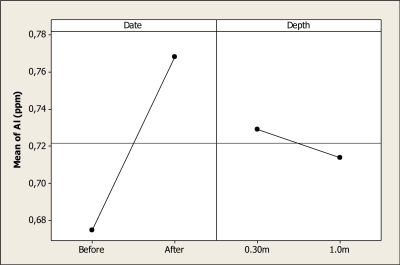
Main effects plot for Al in Colina Lake water samples (Chihuahua, Mexico) before and after the 2010 Holy Week vacation period.

The ANOVA analysis detected differences for Al concentration due to date of sampling (P < 0.001); but no differences were noted for sample depth (P = 0.591) nor their interaction (P = 0.496). The mean Al level before the Holy Week vacation period was 0.67 ppm, while the mean concentration after this period was 0.76 ppm. Figure 2 shows this main effect and domestic tourism is probably contaminating this water reservoir with Al.

The results of this study concerning Al concentration are similar to those values reported by Holguin *et al*. [15], who found levels of 0.91 mgL^–1^ in the lower part of the Conchos River. The Al levels are higher than the Maximum Permitted Level for potable water (0.02 mg L^–1^) established in the Mexican Norm of Ecological Criteria [16]. Unfortunately, there are no criteria concerning Al levels for recreational areas; however, it is evident that the Al concentration is higher after the Holy Week vacation period and, as a consequence, is affecting this recreational ecosystem. It can be hypothesized that garbage like beverage cans and similar objects is responsible for this effect.

**Figure 3.**
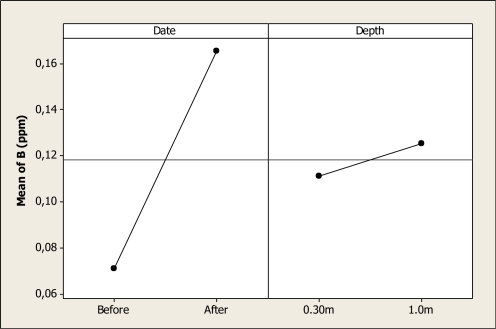
Main effects plot for B in Colina Lake water samples (Chihuahua, Mexico) before and after the 2010 Holy Week vacation period.

The ANOVA detected statistical differences in B levels as a function of sampling date (P < 0.001) but no differences were noted for depth (P = 0.168) or the interaction (P = 0.843). This mean effect is shown in Figure 3, where a maximum B concentration with a mean of about 0.16 ppm was observed after the Holy Week vacation period, in contrast with a mean of 0.07 ppm noted before. Holguin *et al*. [15] reported levels of B of 0.518 mg L^–1^ in the lower part of the Conchos River in the same state, and Colina Lake is situated in the upper part of the Conchos River.

**Figure 4.**
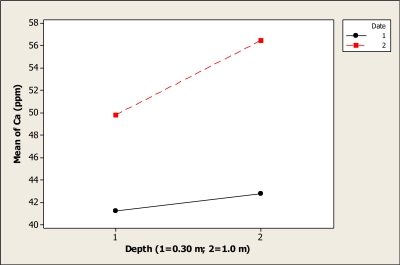
Interaction plot for Ca in Colina Lake water samples (Chihuahua, Mexico) before and after the 2010 Holy Week vacation period.

The ANOVA gave statistical differences in regard to Ca concentration for date (P < 0.001), depth (P < 0.001), and the interaction date-depth (P < 0.004). This interaction effect is shown in Figure 4. Before the tourists visited in the Holy Week vacation period, the Ca concentration was about 41.2 ppm at the 0.30 m depth, and this value increased slightly to about 42.7 ppm at 1.0 m depth. Moreover, the Ca level after the Holy Week period increased at both depths. At 0.30 m depth the Ca concentration was 49.8 ppm and then increased to 56.4 ppm at 1.0 m depth. It is important to mention that there is no specific Maximum Permitted Level for this element in the Mexican norms. Any recreational lake can be classified according to the level of calcium carbonate; for instance, the Grand Lake in the United States of America was classified as moderately hard water lake [17]. Therefore, it is necessary to continue with the monitoring of this lake under study to know more about the different elements like Ca in the water at different seasons. Gutierrez *et al*. [18] found that Ca concentration increased in the lower part of the Conchos River, reaching levels of 120.23 mg L^–1^. These authors also showed that Ca levels increased in the rainy season, and it has to be pointed out that the present study was done in a different season.

**Figure 5.**
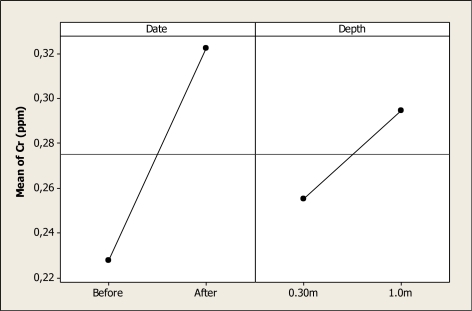
Main effects plot for Cr in water samples of Colina Lake in Chihuahua, Mexico before and after the Holy Week vacation period.

The Cr concentration was statistically different for date (P < 0.000) and depth (P < 0.000), but no differences were discovered for the interaction date-depth (P = 0.554). Figure 5 shows the main factors effect where it is noted that the Cr level increased from 0.22 ppm before the Holy Week vacation period to a level of 0.32 ppm after this period. In addition, it is observed that this element rose from 0.25 ppm at 0.30 m depth to 0.29 ppm at 1.0 m depth. The results in this study are similar to the levels reported previously in the Conchos River during 2005 with a Cr concentration of 0.25 ppm [19]. Nevertheless, in another study carried out in a tributary of the Conchos River, Gutierrez *et al*. [18] reported levels of Cr as higher as 0.014 mg L^–1^; thus, the level of the present study for Cr concentration is higher than previous studies in the same area.

**Figure 6.**
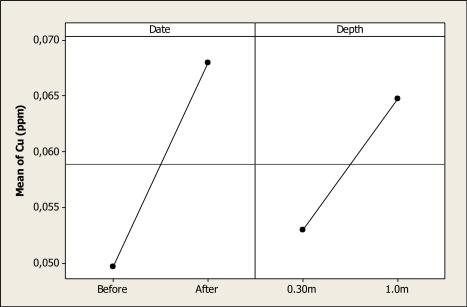
Main effects plot for Cu in Colina Lake water samples (Chihuahua, Mexico) before and after the 2010 Holy Week vacation period.

For Cu, the ANOVA detected significant differences in date (P < 0.001) and depth (P < 0.001), but no differences were noted for the interaction (P = 0.652). The main effect for this element is shown in Figure 6. It can be observed that the mean before the Holy Week vacation period was about 0.04 ppm and this level increased to 0.06 ppm after the vacation period. In addition, considering the depth factor, the mean at 0.30 m depth reached a concentration of 0.05 ppm, and at 1.0 m depth it increased to 0.06 ppm. Cu contamination of water has been considered in some areas to be the major potential threat to drinking water [20], although there exists a controversy concerning the maximum level of copper in water that represents a health risk [21]. A study carried out in 2004 on the Conchos River showed a maximum level of Cu of 0.057 mg L^–1^ in Parra (Chihuahua) which agrees with the findings of the study reported here [22].

**Figure 7.**
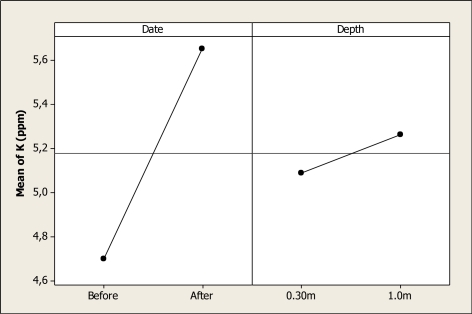
Main effects plot for K in Colina Lake water samples (Chihuahua, Mexico) before and after the 2010 Holy Week vacation period.

Potassium concentration was different as a function of sampling date (P < 0.001) and sampling depth (P = 0.019), and the interaction date-depth was not significant (P = 0.936). Figure 7 shows the main factor effects for this element. It can be seen that before the Holy Week vacation period, the mean concentration was 4.7 ppm, and this level increased to 5.65 ppm after this period. With respect to the depth factor, the mean at 0.30 m depth was 5.08 ppm and 5.26 ppm at 1.0 m depth. Even though a K concentration effect due to human presence in Colina Lake can be noted after the vacation period, this amount does not represent a health risk to local visitors.

**Figure 8.**
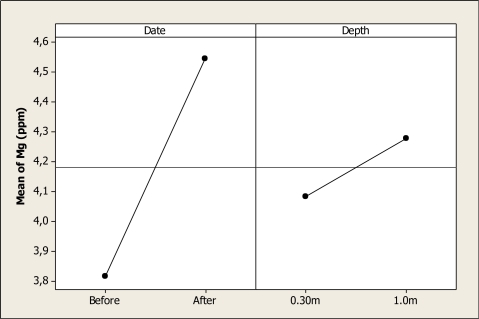
Main effects plot for Mg in Colina Lake water samples (Chihuahua, Mexico) before and after the 2010 Holy Week vacation period.

For Mg, the ANOVA showed significant differences in date (P < 0.001) and for the depth factor (P = 0.007) but no differences were noted for the interaction (P = 0.927). The Mg means before the Holy Week vacation was 3.81 ppm, and after this vacation period it increased to 4.54 ppm (Figure 8). It can also be noted that the mean Mg at 0.30 m depth was 4.08 ppm, and then it increased to a level of 4.27 ppm at 1.0 m depth. The Mg levels reported in this study are lower than those reported previously by Rubio-Arias [22] in a study conducted on six tributaries of the Conchos River.

**Figure 9.**
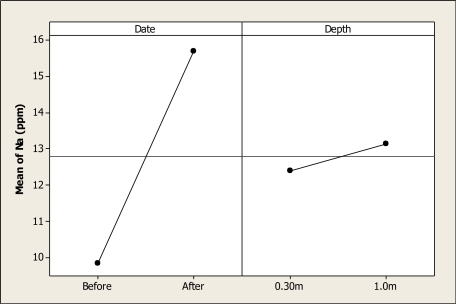
Main effects plot for Na in Colina Lake water samples (Chihuahua, Mexico) before and after the 2010 Holy Week vacation period.

Sodium concentrations were different as a function of date (P < 0.001), but no differences were noted for factor depth (P = 0.065) nor for the interaction (P = 0.928). Figure 9 shows this main effect, and it is noted that before the domestic tourism was present in Colina Lake the amount of Na was about 9.86 ppm. This element increased to 15.70 ppm after the visitors’ stay at the recreational lake. The levels reported in this study for Na were lower than those reported by Holguin *et al*. [15].

**Figure 10.**
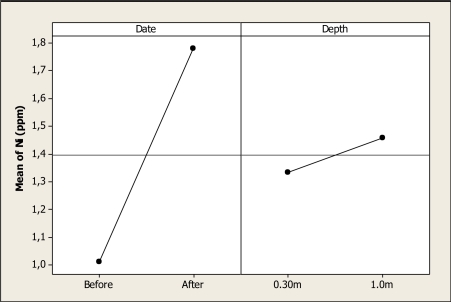
Main effects plot for Ni in Colina Lake water samples (Chihuahua, Mexico) before and after the 2010 Holy Week vacation period.

The Ni concentration was statistically different for date (P < 0.000), but no differences were observed for sampling depth (P = 0.433), nor for the date-depth interaction (P = 0.041). This effect is shown in Figure 10 where the mean of Ni before the Holy Week vacation period was 1.01 ppm, and this element increased to 1.77 ppm after the Holy Week vacation period. The values reported in this study relative to element Ni are higher than previously reported where it was found at 0.47 mg L^–1^ as the most contaminated location [19]. In addition, Ni values are higher than the findings of Holguin *et al*. [15] and Gutierrez *et al*. [18] that were determined in the same watershed. It is important to note that the concentration of this element is higher than those specified as maximum permitted levels for water for agricultural and animal production uses in the Mexican Norm.

**Figure 11.**
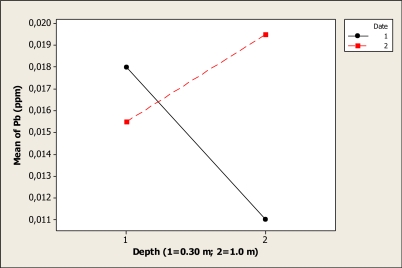
Interaction plot for Pb in Colina Lake water samples (Chihuahua, Mexico) before and after the 2010 Holy Week vacation period.

There were no statistical differences for Pb concentrations for date (P = 0.175) and depth (P = 0.495), but the interaction was slightly significant (P < 0.01). The mean before the Holy Week vacation period was 0.018 ppm at 0.30 m depth, and this level changed to 0.011 ppm at 1.0 m depth (Figure 11). After the vacation period the mean at 0.30 m was 0.015 ppm, and at 1.0 m depth the mean was 0.019 ppm.

**Figure 12.**
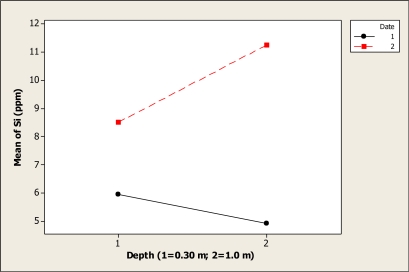
Interaction plot for Si in Colina Lake water samples (Chihuahua, Mexico) before and after the 2010 Holy Week vacation period.

The Si concentration was statistically different for sampling date (P < 0.001), but no differences were noted for depth (P = 0.333). The date-depth interaction was significant (P = 0.01). Figure 12 shows this interaction effect, where it is noted that the mean of Si at 0.30 m depth, before the Holy Week vacation period, was about 5.9 ppm, and then, this level decreased to 4.9 ppm at 1.0 m depth. With respect to after Holy Week period, the Si level was about 8.51 ppm at 0.30 m depth, and then increased to a level of 11.25 ppm at 1.0 m depth.

**Figure 13.**
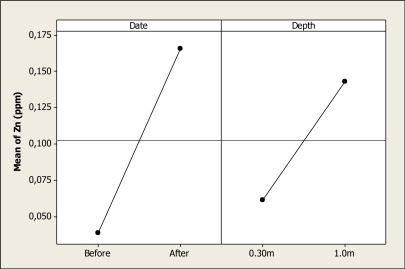
Main effects plot for Zn in Colina Lake water samples (Chihuahua, Mexico) before and after the 2010 Holy Week vacation period.

The Zn concentration was different for sampling date (P < 0.001) and depth (P = 0.01), and the interaction was not significant (P = 0.12). Figure 13 shows the main factor effects where it can be noted that the mean of Zn before the Holy Week vacation period was 0.03 ppm, and then after this period the concentration increased to 0.16 ppm. Also, the mean of this element before the vacation period was 0.06 ppm at a depth of 0.30 m, and this value increased to 0.14 ppm at 1.0 m depth after the vacation period.

## 4. Conclusions

Levels of most heavy metals concentration in this study were higher after the Holy Week vacation period in comparison with the concentration of these elements before the Holy Week vacation period. These results are of practical importance because Colina Lake is obviously being affected by the domestic tourism. Moreover, these results represent a red flag for the authorities as well as for the communities and stakeholders of the area who depend of this recreational area.

## Figures and Tables

**Table 1.** Means and descriptive statistics of the parameters pH, temperature and EC of Colina Lake before and after the Holy Week vacation period.

			**Before**			**After**	
**Parameter**	**Depth (m)**	**Mean**	**SEmean**	**StDev**	**Mean**	**SEmean**	**StDev**
pH	0.30	8.50	0.018	0.084	8.61	0.015	0.069
	1.0	8.53	0.023	0.103	8.56	0.028	0.127
Temperature (°C)	0.30	12.80	0.119	0.532	18.11	0.171	0.763
	1.0	12.16	0.199	0.889	17.75	0.190	0.851
EC (dSm^−1^)	0.30	208	1.380	6.160	228.5	3.420	15.31
	1.0	208	1.170	5.230	226.0	1.520	6.81

**Table 2.** Presence of total coliforms in the water of Colina Lake before Holy Week vacation period.

**SAMPLE**	**DEPTH (m)**	**UFC mL^–1^**	**UFC mL^–1^**	**AVERAGE UFC mL^–1^**
12	0.30	6	0	3
12	1.0	1	0	0.5
13	0.30	8	0	4
13	1.0	0	6	3
19	0.30	1	0	0.5
17	0.30	1	0	0.5
15	0.30	0	2	1
5	0.30	0	3	1.5
18	1.0	0	4	2
10	1.0	2	0	1

**Table 3.** Presence of total coliforms in the water of Colina Lake after Holy Week vacation period.

**SAMPLE**	**UFC mL^−1^**	**UFC mL^−1^**	**MEAN UFC mL^−1^**	**SAMPLE**	**UFC mL^−1^**	**UFC mL^−1^**	**MEAN UFC mL^−1^**
	**DEPTH (1.0 m)**				**DEPTH (0.30 m)**		
18	0	2	1	9	0	1	0.5
5	1	0	0.5	17	0	3	1.5
18	2	0	1	19	0	1	0.5
14	0	2	1	2	0	3	1.5
10	0	2	1	9	0	1	0.5
10	0	2	1	15	0	2	1
19	0	5	2.5	13	0	2	1
19	0	12	6	20	0	2	1
8	0	4	2	6	0	7	3.5	
20	0	1	0.5	16	0	3	1.5	
9	0	6	3	19	0	1	0.5	
6	0	5	2.5	14	0	1	0.5	
5	0	5	2.5	8	0	8	4	
16	0	6	3	8	0	6	3	
16	0	6	3	6	0	3	1.5	
10	0	1	0.5	16	0	3	1.5	
3	0	5	2.5	4	0	2	1	
4	0	6	3	17	0	3	1.5	
6	0	4	2					
15	0	5	2.5					
3	0	1	0.5					
9	0	5	2.5					
8	0	5	2.5					
13	0	1	0.5					

**Table 4.** Presence of fecal coliform in the water of Colina Lake after the Holy Week vacation period.

**SAMPLE**	**DEPTH (m)**	**UFC mL^−1^**	**UFC mL^−1^**	**AVERAGE UFC mL^−1^**
19	0.30	6	0	3
19	0.30	3	0	1.5
4	1.0	4	0	2
9	1.0	2	0	1
14	1.0	3	0	1.5
15	1.0	1	0	0.5
2	0.30	0	2	1
6	1.0	3	0	1.5
16	0.30	2	0	1
16	0.30	3	0	1.5
8	0.30	1	0	0.5
15	0.30	1	0	0.5
11	0.30	1	0	0.5
18	1.0	1	0	0.5
10	1.0	2	0	1
10	1.0	5	0	2.5
4	1.0	3	0	1.5
10	0.30	3	0	1.5
